# Adherence to the Mediterranean Diet Has a Protective Role against Metabolic and DNA Damage Markers in Colorectal Cancer Patients

**DOI:** 10.3390/antiox11030499

**Published:** 2022-03-04

**Authors:** Delia Acevedo-León, Segundo Ángel Gómez-Abril, Lidia Monzó-Beltrán, Nuria Estañ-Capell, Rafael Arroyo-Montañés, Celia Bañuls, Jordi Salas-Salvadó, Guillermo Sáez

**Affiliations:** 1 Service of Clinical Analysis, University Hospital Dr. Peset, Foundation for the Promotion of Health and Biomedical Research in the Valencian Region (FISABIO), 46017 Valencia, Spain; acevedo_del@gva.es (D.A.-L.); estany_nur@gva.es (N.E.-C.); arroyo_raf@gva.es (R.A.-M.); 2 Department of General Surgery and Digestive System, University Hospital Dr. Peset, Foundation for the Promotion of Health and Biomedical Research in the Valencian Region (FISABIO), 46017 Valencia, Spain; gomez_seg@gva.es; 3 Department of Biochemistry and Molecular Biology, Faculty of Medicine and Odontology, Health Research Institute, Clinical Hospital of Valencia, University of Valencia, 46010 Valencia, Spain; limonbel@alumni.uv.es; 4 Department of Endocrinology and Nutrition, University Hospital Doctor Peset, Foundation for the Promotion of Health and Biomedical Research in the Valencian Region (FISABIO), 46017 Valencia, Spain; 5 Unitat de Nutrició Humana, Departament de Bioquímica i Biotecnologia, Universitat Rovira i Virgili, 43201 Reus, Spain; jordi.salas@urv.cat; 6 Institut d’Investigació Sanitària Pere Virgili (IISPV), Hospital Universitari San Joan de Reus, 43204 Reus, Spain; 7 Consorcio CIBER, M.P. Fisiopatología de la Obesidad y Nutrición (CIBERObn), Instituto de Salud Carlos III (ISCIII), 28029 Madrid, Spain

**Keywords:** colorectal cancer, Mediterranean diet, oxidative stress, 8-oxodG, antioxidants

## Abstract

Oxidative stress (OS) and inflammation have been related to colorectal cancer (CRC), but the influence of the Mediterranean diet (MD) on these parameters is unknown. Therefore, the aim of this study was to determine the association between adherence to the MD and markers of OS and DNA damage in CRC patients and to study the influence of adherence to the MD on metabolic and tumor-related factors. This prospective observational study included a total of 80 patients diagnosed with CRC. Adherence to the MD was estimated by the 14-item Mediterranean Diet Adherence Screener (MEDAS) questionnaire. The levels of OS markers (catalase, glutathione peroxidase, and glutathione system in serum; 8-oxo-7′8-dihydro-2′-deoxyguanosine and F2-isoprotanes in urine) and tumor and metabolic factors were determined. A total of 51.2% of our CRC patients showed a high adherence to the MD. These patients presented decreased levels of 8-oxodG, increased GPX and HDL–cholesterol levels, and a downward trend in the GSSG/GSH ratio with respect to patients with low adherence to the MD. In addition, a high adherence to the MD was associated with a lower histological grade of the tumor and a lower presence of synchronous adenomas. We conclude that a high adherence to the MD has a protective role against metabolic and oxidative DNA damage and improves antioxidant systems in CRC patients.

## 1. Introduction

Oxidative stress (OS), chronic inflammation and cancer are closely related. There is increasing evidence that chronic inflammation and associated OS induce DNA damage, thereby promoting a variety of human cancers [1]. Reactive oxygen species are potential carcinogens, as they can cause DNA oxidation and alterations in the expression of tumor suppressor genes and proto-oncogenes [2], thus favoring mutagenesis and tumor promotion and progression. An impaired redox status combined with an excessive production of reactive oxygen/nitrogen species has been implicated in several cancers and diseases, including the development and progression of colorectal cancer (CRC) [3].

CRC, one of the most common cancers in many developed countries, has been associated with multiple environmental and lifestyle factors (e.g., obesity, dietary patterns, and physical inactivity) [4] and is marked by high OS leading to genomic instability.

Elevated OS is highly prominent in CRC, as evidenced by the presence in clinical samples of increased levels of oxidized DNA bases, such as 8-oxo-7′8-dihydro-2′-deoxyguanosine (8-oxodG), and lipid peroxidation products [5,6]. In addition, altered levels of ROS-scavenging enzymes, such as superoxide dismutase, glutathione peroxidase (GPX), and peroxiredoxin, are indicative of aberrant redox homeostasis in tumor cells [2,7].

The primary prevention measure for CRC in our country (Spain) consists of screening strategies to detect blood in stools and information campaigns to promote healthy habits and lifestyles in the general population in order to decrease the risk of developing the disease. The Mediterranean diet (MD) is characterized by a high intake of olive oil and plant-based foods (fruits, vegetables, legumes, nuts, and whole cereals), a moderate intake of fish, poultry, dairy products, and alcohol, and a low intake of red meat, processed food, and sweets [8]. The importance of the MD in terms of health is not limited to its balance, with a low content of saturated fatty acids and a high content of monounsaturated fatty acids and fiber, but is also due to an abundance in antioxidant substances, polyphenols, phytosterols, and other beneficial substances of vegetable origin. Most of these foods are rich sources of bioactive compounds that seem to play a synergistic role in protecting against OS, including DNA damage [9]. Moreover, this diet has been shown to have a preventive effect with respect to diabetes, other cardiometabolic diseases [10,11], as well inflammatory diseases [10] and cancer [12], including CRC [13].

Adherence to the MD has been inversely related to coronary heart disease mortality [14], cancer mortality, and all-cause mortality [15]. In addition, there is much evidence that the traditional MD is associated with a reduced risk of other major chronic diseases [10,11,12,16,17]. However, little research has been carried out to explore the role of the MD in the modulation of OS and DNA damage as potential contributors to CRC.

Therefore, the objective of our study was to determine the association between adherence to the MD and levels of OS and oxidative damage markers in patients with CRC and thus identify potentially preventive factors in this disease. In addition, the association between adherence to MD and several metabolic markers, tumor-related factors, and tumor stage was studied.

## 2. Materials and Methods

This was a cross-sectional observational study of 80 patients with CRC, with indication for tumor resection surgery and/or chemoradiotherapy. The patients had been referred to the General and Digestive Surgery Service of University Hospital Dr. Peset in Valencia, Spain, and were sequentially enrolled into the study. The study was designed in accordance with the ethical principles of the Declaration of Helsinki (Finland, 1964) and approved by the Clinical Research Ethics Committee of University Hospital Dr. Peset. Written informed consent was obtained from all participants in the study. 

Analytical determinations were carried out in the hospital’s Clinical Analysis Laboratory and in the Department of Biochemistry and Molecular Biology of the Faculty of Medicine and Odontology of the University of Valencia. 

Patients completed the validated MEditerranean Dietary Adherence Screener (MEDAS) questionnaire to measure their adherence to the MD [18]. The test is a brief dietary assessment instrument consisting of a set of 14 short questions about daily or weekly consumption of different food groups (see Supplementary Table S1). Briefly summarized, the questions refer to the following (question number): olive oil (1 and 2); vegetables (3); fruit (4); red meat, sausages, and other processed meat (5); butter, margarine, or cream (6); carbonated and/or sugary drinks (7); wine (8); legumes (9); fish or seafood (10); industrially produced pastries (not homemade) (11); nuts (12); chicken, turkey, or rabbit vs. beef or pork (13); sofrito, a sauce that includes tomato, onion, garlic, and olive oil (14). Adherence was considered high when the MEDAS score was equal to or greater than 9 [19].

Anthropometric variables, blood pressure, data related to colorectal tumor (TNM stage, tumor size, location, association of adenomatous polyps, diverticulosis, etc.), clinical data, and the presence of comorbidities of interest (obesity, hypertension, diabetes mellitus, and dyslipidemia) were recorded.

The following OS markers were evaluated: catalase (CAT), GPX, and glutathione system in serum; 8-oxodG and F2-isoprotans in urine. The first urine specimen of the day was collected, and so urinary creatinine excretion was also determined by spectrophotometry as a correction factor for the concentration or dilution of the sample. 

Cayman Chemical (Ann Arbor, MI, USA) assays were used for CAT, GPX, the glutathione system (by spectrophotometry), and F2-Isoprostanes (by ELISA). The manufacturer’s methodological protocol was followed, with the subsequent reading of absorbance by a Thermo Labsystems Multiskan EX spectrophotometry/ELISAS reader (Beverly, MA, USA).

The molecule 8-oxodG was determined by high-resolution chromatography with an ESA Coulochem II electrochemical HPLC detector (Hucoa-Erlöss, Madrid, Spain). All sample dilutions, reagent preparations, and material washings were performed with HPLC-grade-purity water (ultrapure or type I).

In addition to OS markers, serum biochemical (glucose, cholesterol, and fractions, triglycerides, uric acid, and albumin) and tumor (CEA and CA 19.9) markers were measured. Biochemical determinations were performed by spectrophotometry in the automated chain of an Architect C16000 equipment from Abbott (Chicago, IL, USA) and the tumor markers were determined by electrochemiluminescence using a Cobas 6000 from Roche Diagnostics (Mannheim, Germany). 

In order to perform statistical analyses of the association of the different variables with tumor stage, the TNM stages of the American Joint Committee on Cancer (AJCC) were transformed into qualitative variables 0, 1, and 2 in similar to those of the American Cancer Society (ACS) [20], which is based on information from the Surveillance, Epidemiology, and End Results (SEER) database of the National Cancer Institute (NCI): tumors located in the colon/rectum, including TNM 0, I, and II stages = Stage 0, regional tumors involving lymph nodes, including TNM III stage = Stage 1, and advanced tumors, with metastasis or invasion in distant sites of the peritoneum, including TNM IV stage = Stage 2.

For statistical analysis, SPSS 17.0 (Chicago, IL, USA) was used, and parametric and non-parametric statistical tests were applied depending on the homogeneity of the variance and the normality of the variables: Student’s T and Mann–Whitney’s U were used for quantitative variables, and Pearson’s χ² test for the comparison of qualitative variables. For the study of the different correlations between the variables, a bivariate correlation with P of Pearson was applied to parametric-distribution variables, and Spearman’s Rho was applied to non-parametric variables. The statistical significance level used in all cases was *p* < 0.05.

## 3. Results

The demographic characteristics and anthropometric and biochemical variables of the CRC patients recruited for this study are shown in Table 1, distributed according to their adherence to the MD. Mean age and body mass index were 67.5 ± 11.8 years and 28.0 ± 3.9 Kg/m^2^, respectively, and 65% of the cohort were men. No significant differences were found in any of the anthropometric and biochemical parameters in relation to adherence to the MD, with the exception of HDL–cholesterol, which was higher in patients with high adherence (*p* = 0.048).

The mean MEDAS questionnaire score of the 80 recruited CRC patients was 8.6, out of a maximum total of 14 points. We found that 51.25% of the patients (*n* = 41) showed high adherence to the MD, with a mean score of 10.5 compared to a score of 6.7 for patients with low adherence. The items most commonly receiving a positive answer by the patients were numbers 1, 6, 7, and 13, concerning the consumption of olive oil for cooking and dressing, dairy product consumption, low consumption of sugary drinks, and consumption of white meat rather than red meat (85%), followed by item 2, related to the use of olive oil as the main oil used (81.3%), item 5, on the intake of red meat (73.8%), item 14, referring to dishes seasoned with olive oil (71.3%), item 11, on the consumption of industrially manufactured pastries (62.5%), item 4, fruits (60%), item 12, nuts (50%), item 10 and 3, related to the consumption of fish and vegetables (33.8%), item 9, regarding the intake of legumes (28.8%), and finally item 8, related to wine consumption (25%).

Regarding the presence of comorbidities, no significant differences were found between the participants with high adherence to the MD and those with low adherence to the MD for the prevalence of obesity (60.7% vs. 35.7%), hypertension (48.7% vs. 51.3%), dyslipidemia (48.8% vs. 51.2%), or diabetes (45.2% vs. 54.8%).

In reference to the tumor markers used in the diagnosis of CRC, patients with a high adherence to the MD showed lower levels of CEA and CA 19.9 than those with low adherence, although the differences were not significant (Table 1). 

In relation to the tumor stage, tumors at stages 0, 1, and 2 were found in 61%, 34.1%, and 4.9%, respectively, of high-adherence subjects, compared to 47.4%, 36.8%, and 15.8% of patients with low adherence, although no significant differences were observed between the groups (Table 2). An association was found between adherence to the MD and the WHO histological grade of the tumor. Among patients with low-grade tumors, 56.5% of them showed a high adherence to the MD, while 43.5% reported a low adherence to the MD, and only 20% of patients with a high histological grade tumor showed high adherence to the MD whereas 80% reported low adherence (*p* = 0.031). A similar pattern was observed for patients also presenting adenomas, which were less frequently observed in high-adherence patients (38.9%) than in patients who reported low adherence to the MD (61.1%). 

**Figure 1:**
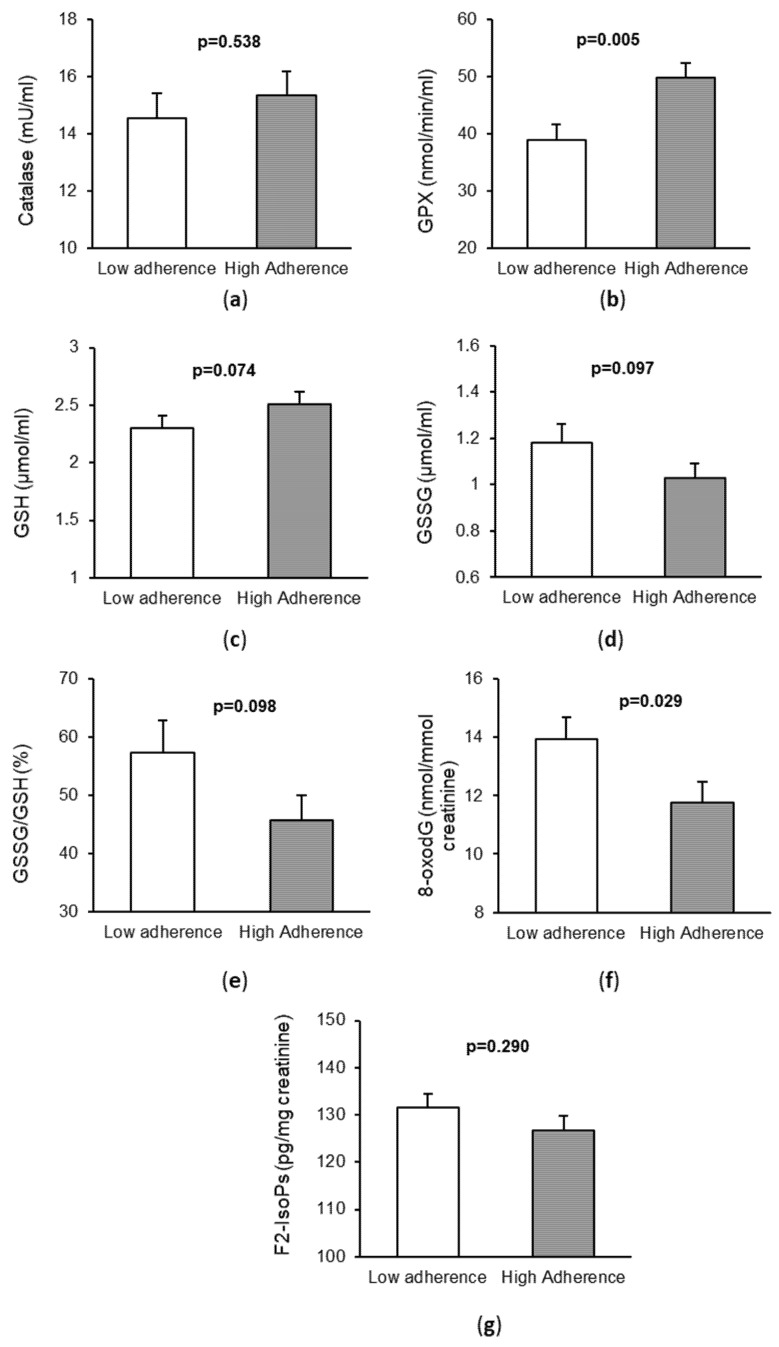
Markers of oxidative stress and DNA damage in patients with colorectal cancer according to their adherence to the Mediterranean diet. (**a**) Catalase activity in serum; (**b**) Glutathione peroxidase (GPX) activity in serum; (**c**) Levels of Reduced Glutathione (GSH) in serum; (**d**) Levels of Oxidized Glutathione (GSSG) in serum; (**e**) Ratio GSSG/GSH in serum; (**f**) Levels of: 8-oxo-7,8-dihidro-2′-deoxyguanosine 8-oxodG (8-oxodG) in urine; (**g**) Levels of F2-isoprostanes (F2-IsoPs) in urine.

Regarding the markers of OS and oxidative damage in serum or urine, we observed that patients with high adherence to the MD had higher levels of the serum antioxidant GPX (*p* = 0.029) and lower levels of urine 8-oxodG (*p* = 0.005), as shown in Figure 1. In addition, in CRC patients with high adherence to the MD, there was a trend towards higher levels of reduced glutathione (GSH, *p* = 0.074), lower levels of oxidized glutathione (GSSG, *p* = 0.097), and lower GSSG/GSH ratio (*p* = 0.098) with respect to those with low adherence to the MD. However, no significant differences were observed for CAT (*p* = 0.538) and F2-isoprostanes (*p* = 0.290) between the two groups. 

When we analyzed the correlations between MEDAS score and levels of tumor and OS markers (Table 3), a positive correlation with GPX was detected (*p* = 0.045). In addition, CEA concentrations negatively correlated with the levels of CAT (*p* < 0.001), GPX (*p* < 0.001), and GSH (*p* = 0.005) and positively correlated with the levels of GSSG (*p* < 0.001), the GSSG/GSH ratio (*p* < 0.001), and the levels of 8-oxodG (*p* = 0.009) and F2-isoprostanes (*p* = 0.015). On the other hand, CA 19.9 levels negatively correlated with CAT (*p* < 0.001), GPX (*p* = 0.005), and GSH (*p* = 0.022) and positively correlated with GSSG levels (*p* < 0.001), the GSSG/GSH ratio (*p* = 0.002), and F2-isoprostanes levels (*p* = 0.001).

## 4. Discussion

The present study provides evidence that adherence to the MD is associated with an increase in HDL–cholesterol and GPX levels and a decrease in 8-oxoDG levels. In relation to the adherence to the MD, we detected significant correlations between tumor and OS markers. Adherence to the MD also provided protection in terms of histological grade and stage of the tumor. 

With regard to the 14 items that make up the MEDAS questionnaire, over 85% of patients complied with 5 of them, while the percentage was below 85% for 9 items. Of note was the high compliance with the use of olive oil for cooking and dressing food, the dairy product consumption, the low consumption of sugary drinks, and the consumption of white rather than red meat. When we compared our results with those of studies in which the same screening method was used, the mean score of our cohort was similar to the baseline score of the PREDIMED study [21] and higher than that of general-population surveys [22]. A total of 51.3% of our patients showed high adherence to the MD (score ≥ 9), which was also similar to the baseline percentage of the PREDIMED study. 

The PREDIMED Spanish trial reported that an intervention with a vegetable-based MD rich in unsaturated fat and polyphenols had preventive effects against cardiovascular disease [21,23]. It is widely recognized that MD has beneficial effects on cardiometabolic health, including blood pressure, insulin sensitivity, and lipid profile, thus decreasing the risk of cardiovascular disease [10,24,25]. Regarding the biochemical parameters, we observed significant differences in lipid profile, especially in HDL–cholesterol levels, which were higher among participants with high adherence to the MD. Several studies evaluated the association between adherence to the MD and HDL–cholesterol levels [10,26,27,28], a recognized risk factor for cardiovascular disease. Dietary polyphenol intake has been shown to increase serum HDL–cholesterol levels and may represent a potential treatment to raise HDL–cholesterol in dyslipidemic subjects [26]. The PREDIMED study also showed a reversion of metabolic syndrome due to beneficial effects on HDL–cholesterol, and also on other cardiovascular risk factors related to the syndrome [27]. Moreover, MD has been demonstrated to improve HDL–cholesterol function in high-cardiovascular-risk individuals [28]. 

The consumption of red meat (beef, pork, lamb, veal, mutton) is high in developed countries and is considered probably carcinogenic for humans [29]. Different mutagenic and/or carcinogenic compounds may explain the relationship between red meat consumption and CRC. These include heme iron in red meat, N-nitroso compounds, heterocyclic amines, polycyclic aromatic hydrocarbons, polyunsaturated fatty acids, bile acids, non-human sialic acid, and infectious agents [30,31]. In fact, accumulated evidence of prospective epidemiological studies shows that the consumption of red meat and processed meat is a risk factor for developing polyps and CRC [32] and increases the CRC risk by 20–30% [33]. The results of a large number of investigations have corroborated the health benefits of significantly reducing the consumption of red meat [34,35,36]. Hence, the MD plays a protective role by containing a low amount of red and processed meat.

Several reviews and meta-analyses in observational studies conducted in different cohorts with different types of cancer have highlighted an inverse association between a high adherence to the MD and the initiation and progression of cancer [13], especially in the case of CRC [30,37,38]. For example, olive oil supplementation has been shown to be inversely associated with CRC risk [39]. We observed a lower presence of synchronous adenomas in CRC patients with high adherence to the MD. In accordance with our results, Zheng et al. reported that the MD was inversely associated with the risk of early-onset adenoma [40]. An inverse association between adherence to the MD and the WHO histological grade of the tumor was also found. To the best of our knowledge, no other study has been conducted to analyze this potential association. 

On the other hand, we found no association with tumor stage, although 61% of our participants with high adherence to the MD were in stage 0, and only 4.9% were in stage 2. In the same way, in a prospective cohort study of patients with metastatic CRC, a non-significant inverse association between adherence to the MD and risk of death was reported [41].

CRC is related to disturbances of antioxidant defenses and increased oxidative and nitrosative damage to proteins and DNA [6,42]. It is generally considered that, at higher levels of OS, the levels of GPX and GSH decrease, and those of GSSG, the GSSG/GSH ratio, and 8-oxodG and F2-isoprostanes levels increase. In addition, a key role of cellular pathways that provide protection against OS is played by vitagenes, including heat shock proteins as well as the thioredoxin/thioredoxin reductase system [43]. In this context, dietary antioxidants have been shown to be neuroprotective through the activation of hormetic pathways. It has been shown that under systemic OS conditions, the induction of Hsp70 vitagenes is a maintained response to offset the intracellular pro-oxidant status triggered by the reduction of GSH content and thioredoxin expression [44]. Different studies have shown that the MD significantly reduces the levels of a wide range of biomarkers of different aspects of OS, including those indicating oxidative DNA damage [45,46,47,48,49], lipid peroxidation, reactive metabolic products and by-products [47,50], and endogenous immune-inflammatory activation as a source of OS [46,51]. In contrast, higher levels of biomarkers of antioxidant defense and ROS detoxification have been reported [52]. MD adherence has been associated with increased levels of the antioxidant GPX and inversely correlated with the levels of F2-isoprostanes [53]. In accordance with previous studies [3,42], our results show that the antioxidant levels negatively correlate with tumor-related markers and that the levels of oxidative products positively correlate with different tumor-related markers.

Regarding the influence of the MD on OS, Dai et al. [17] reported that a one-unit increment in the MD score described by Trichopoulou et al. [22] was associated with a 7% higher GSH/GSSG ratio and lower OS. In our study, the serum levels of GPX positively correlated with the MEDAS score. These results can be considered important, since low levels of this enzyme have been related to an increase in cardiovascular risk [53,54]. Traditional Mediterranean dietary patterns, as well as other healthy diets, may reduce the plasma levels of inflammatory factors and OS markers [55]. We observed a trend towards more pronounced levels of markers of OS in patients with low adherence to the MD, although the differences were significant only for 8-oxodG and GPX. Luisi et al. [46] studied the effect of an MD rich in extra virgin olive oil in individuals with obesity and found a decline in OS markers (8-oxodG) in both obese and control subjects. Poor adherence to the MD has also been associated with impaired OS in other metabolic conditions, such as non-alcoholic fatty liver disease [56]. In addition, published studies reported a reduction in the levels of 8-oxodG and a modulation of DNA repair gene expression [9]. In particular, three studies reported a decrease in 8-oxo-dG levels in urine [57], plasma [58], stools [59], or peripheral blood leukocytes [60] after an MD intervention alone or in combination with other food/dietary components, while only one study failed to report significant effects [61]. Conversely, one study reported a significant reduction in the levels of oxidatively induced, but not endogenous, DNA damage [62]. These discrepancies between the different OS markers may be due to the fact that not all these markers are equivalent and that they do not equally respond to specific dietary interventions and clinical conditions.

## 5. Conclusions

Compared to those with low adherence to the MD, patients with high adherence displayed lower levels of 8-oxodG and increased concentrations of HDL–cholesterol and GPX. Adherence to the MD was associated with the presence of a low WHO tumor histological grade and a lower presence of synchronic adenomas, all of which are factors of good prognosis.

The results of our study suggest that adherence to the MD has a protective role in CRC patients, although it is necessary to confirm these findings in a larger sample of CRC patients in a prospective study.

## Figures and Tables

**Table 1:** Demographic, anthropometric, and biochemical variables of patients with colorectal cancer according to their adherence to the Mediterranean diet.

	Total (*n* = 80)	Low Adherence to MD (*n* = 39)	High Adherence to MD (*n* = 41)	*p*-Value
Age (years) Male/female (%) BMI (Kg/m^2^)	67.5 ± 11.8	66.1 ± 12.5	68.9 ± 11.2	0.297
	35.0/65.0	66.7/33.3	63.4/36.6	0.764
	28.0 ± 3.9	28.0 ± 3.7	28.1 ± 4.2	0.891
Glucose (mg/dL) Total cholesterol (mg/dL)	116.6 ± 52.3	116.3 ± 40.6	116.9 ± 30.2	0.943
	180.5 ± 39.1	181.3 ± 36.0	179.6 ± 42.2	0.850
HDLc (mg/dL) LDLc (mg/dL)	43.2 ± 10.8	40.8 ± 9.8	45.5 ± 11.4	0.048
	114.5 ± 34.7	115.7 ± 33.6	113.3 ± 36.2	0.762
Triglycerides (mg/dL)	108.5 (83.3; 141.0)	109.0 (83.0; 150.0)	106.0 (81.5; 133.5)	0.185
Uric acid (mg/dL)	5.3 ± 1.7	5.1 ± 1.8	5.5 ± 1.6	0.282
Albumin (g/dL)	4.2 ± 0.5	4.2 ± 0.3	4.2 ± 0.6	0.505
CEA (ng/mL)	2.9 (1.7; 6.0)	3.5 (1.9; 11.6)	2.0 (1.6; 4.7)	0.081
CA 19.9 (IU/mL)	13.3 (6.3; 26.4)	15.5 (7.9; 32.4)	9.4 (5.7; 24.0)	0.164

Abbreviations. MD: Mediterranean diet; HDLc: high-density lipoprotein cholesterol; LDLc: low-density lipoprotein cholesterol; CEA: carcinoembryonic antigen CA 19.9: carbohydrate antigen 19.9. Data are expressed as mean ± SD for parametric data and median (interquartile range) for non-parametric variables.

**Table 2:** Relationship between patients’ adherence to the Mediterranean diet and factors related to their CRC tumor.

(%)	Low Adherence to MD	High Adherence to MD	*p*-Value
Tumor diameter <6 (*n* = 67) ≥6 (*n* = 9)			0.923
	47.8	52.2	
	44.4	55.5	
WHO histological grade			0.031
Low (*n* = 69)	43.5	56.5	
High (*n* = 10)	80.0	20.0	
Co-existence of adenomas			0.034
No (*n* = 44)	37.2	62.8	
Yes (*n* = 36)	61.1	38.9	
Presence of diverticulosis			0.203
No (*n* = 65)	51.6	48.4	
Yes (*n* = 15)	33.3	66.7	
Tumor localization			0.137
Right colon (*n* = 29)	34.5	65.5	
Left colon (*n* = 23)	60.9	39.1	
Rectum (*n* = 28)	53.6	46.4	
Synchronous tumors			0.949
No (*n* = 74)	48.6	51.4	
Yes (*n* = 6)	50.0	50.0	
Complications *			0.270
No (*n* = 61) Yes (*n* = 15)	49.2	50.8	
	33.3	66.7	
Tumor recurrence			0.926
No (*n* = 74)	46.7	53.3	
Yes (*n* = 2)	50.0	50.0	
Tumor stage			0.220
0 (*n* = 45)	47.4	61.0	
1 (*n* = 26)	36.8	34.1	
2 (*n* = 9)	15.8	4.9	

* Include post-operative complications (ileus, bleeding, suture dehiscence, and occlusions). MD: Mediterranean Diet.

**Table 3:** Correlation analysis between adherence to the Mediterranean diet, oxidative stress, and tumor markers.

	Adherence to MD Score (MEDAS)	CEA	CA 19.9
CAT serum GPX serum GSH serum	r = 0.004; *p* = 0.974	r = −0.398; *p* < 0.001	r = −0.462; *p* < 0.001
	r = 0.225; *p* = 0.045	r = −0.352; *p* = 0.001	r = −0.311; *p* = 0.005
	r = 0.141; *p* = 0.211	r = −0.309; *p* = 0.005	r = −0.256; *p* = 0.022
GSSG serum GSSG/GSH serum	r = −0.118; *p* = 0.299	r = 0.461; *p* < 0.001	r = −0.459; *p* < 0.001
	r = −0.114; *p* = 0.313	r = 0.429; *p* < 0.001	r = 0.349; *p* = 0.002
8-oxodG urine F2-IsoPs urine	r = −0.163; *p* = 0.148	r = 0.291; *p* = 0.009	r = 0.170; *p* = 0.132
	r = 0.008; *p* = 0.942	r = 0.271; *p* = 0.015	r = 0.367; *p* = 0.001

Abbreviations. MD: Mediterranean Diet; CEA: carcinoembryonic antigen; CA 19.9: carbohydrate antigen 19.9.CAT: Catalase; GPX: Glutathione peroxidase; GSH: Reduced Glutathione; GSSG: Oxidized Glutathione; 8-oxodG: 8-oxo-7,8-dihidro-2′-deoxyguanosine; F2-IsoPs: F2-Isoprostanes.

## Data Availability

All of the data is contained within the article and the supplementary materials.

## Supplementary Materials

The following are available online at https://www.mdpi.com/article/10.3390/antiox11030499/s1, Table S1: MEditerranean Dietary Adherence Screener (MEDAS) questionnaire.
