# Supplementary material for: Adherence to the Mediterranean Diet Has a Protective Role against Metabolic and DNA Damage Markers in Colorectal Cancer Patients
Source: Antioxidants (Basel). 2022 Mar 4;11(3):499. doi: 10.3390/antiox11030499 (PMC8944597; doi:10.3390/antiox11030499)
Supplement: Supplementary file 1 [file antioxidants-11-00499-s001.zip › antioxidants-1609695-supplementary.pdf]

Supplementary Table S1

	QUESTION	Assessment
1	Do you use olive oil as the principal source of fat for cooking?	1 point given: yes
2	How much olive oil do you consume per day (including that used in frying, salads, meals eaten away from home, etc.)?	1 point given if ≥4 Tbsp
3	How many servings of vegetables do you consume per day? Count garnish and side servings as ½ point; a full serving is 200g.	1 point given if ≥2
4	How many pieces of fruit (including fresh-squeezed juice) do you consume per day?	1 point given if ≥3
5	How many servings of red meat, hamburgers, or sausages do you consume per day? A full serving is 100-150g.	1 point given if <1
6	How many servings (12g) of butter, margarine or cream do you consume per day?	1 point given if <1
7	How many carbonated and/or sugar-sweetened beverages do you consume per day?	1 point given if <1
8	Do you drink wine? How much do you consume per week?	1 point given if ≥7 cups
9	How many servings (150 g) of pulses do you consume per week?	1 point given if ≥3
10	How many servings of fish /seafood do you consume per week? (100-150 g o fish, 4-5 pieces or 200 g of seafood)	1 point given if ≥3
11	How many times do you consume industrially produced (not homemade) pastries (such as cookies or cake) per week?	1 point given if <2
12	How many times do you consume nuts per week? (1 serving = 30 g)	1 point given if ≥3
13	Do you prefer to eat chicken, turkey or rabbit instead of beef, pork, hamburgers or sausages?	1 point given: yes
14	How many times per week do you consume boiled vegetables, pasta, rice, or other dishes with a sauce of tomato, garlic, onion, or leeks sautéed in olive oil?	1 point given if ≥2
